# High Prevalence of Multidrug-Resistant and Extended-Spectrum *β*-Lactamase-Producing Enterobacteriaceae: A Cross-Sectional Study at Arsho Advanced Medical Laboratory, Addis Ababa, Ethiopia

**DOI:** 10.1155/2020/6167234

**Published:** 2020-04-30

**Authors:** Adane Bitew, Estifanos Tsige

**Affiliations:** ^1^Department of Medical Laboratory Sciences, College of Health Sciences, Addis Ababa University, Addis Ababa, Ethiopia; ^2^Department of Microbiology and Molecular Biology, Arsho Advanced Medical Laboratory, Addis Ababa, Ethiopia

## Abstract

**Background:**

Multidrug-resistant Enterobacteriaceae particularly extended-spectrum beta-lactamase producers have become a major public health threat. Despite efforts to limit their spread, rates of multidrug-resistance members of the Enterobacteriaceae continue to increase throughout the world causing increased morbidity and mortality and raised costs for medical care.

**Objective:**

The aim of this study was to determine the prevalence of multidrug resistance and extended-spectrum *β*-lactamase-producing Enterobacteriaceae.

**Methods:**

Four hundred forty Enterobacteriaceae isolates from outpatients referred to Arsho Advanced Medical Laboratory were identified and assessed for their antimicrobial resistance pattern by using the automated VITEK 2 compact system. Extended-spectrum *β*-lactamase production was determined by the VITEK 2 automated compact system using the extended-spectrum *β*-lactamase test panel as per the instruction of the manufacturer.

**Results:**

The overall resistance rates of Enterobacteriaceae against cephalosporins, aminoglycosides, and fluoroquinolones were high. Nitrofurantoin with a resistance rate of 14.3% and piperacillin/tazobactam combination with a resistance rate of 17.3% were better active against this group of Gram-negative bacteria. Out of 440 isolates of Enterobacteriaceae, 42.1% were multidrug-resistant of which 34.3% and 8.95% were extensively drug-resistant and pan-drug resistant, respectively. Among 185 multidrug-resistant Enterobacteriaceae, 63.9% of the isolates produced extended-spectrum *β*-lactamase of which 75.4%, 19.5%, 1.7%, 2.5%, and 0.8% were *E. coli*, *K. pneumoniae*, *C*. *freundii*, *E*. *cloacae*, and *P*. *mirabilis*, respectively.

**Conclusions:**

The present study demonstrated high prevalence rates of multidrug-resistant and extended-spectrum-beta-lactamase-producing Enterobacteriaceae. In order to combat these problems, infection control strategy and proper antibiotic policies should be formulated.

## 1. Background

Enterobacteriaceae is a large family consisting of Gram-negative bacteria implicated in causing a variety of human diseases, such as urinary tract infections, wound infections, gastroenteritis, meningitis, septicemia, and pneumonia [1, 2].

An increase in the level of antimicrobial resistance among Gram-negative bacteria in the family Enterobacteriaceae has remained a growing public health threat across the globe. Acquisition and transferring of drug resistance genes to the same or different species of bacteria through mobile genetic elements such as plasmids and transposons are the main properties of most members of the family. Drug resistance genes so acquired are known to facilitate bacteria to produce *β*-lactamase enzymes particularly extended-spectrum *β*-lactamase (ESBL) that confer resistance to the majority of *β*-lactam antibiotics [1, 3–6]. Carbapenems have been used as the latest alternative to treat infections caused by multidrug-resistant (MDR) Enterobacteriaceae [7, 8], but with no satisfactory success, as there has been a development of carbapenem-resistant Enterobacteriaceae worldwide [1]. Enterobacteriaceae have also been identified to have additional drug resistance mechanisms to other categories of antimicrobials such as phenicols, sulfonamides, fluoroquinolones, tetracyclines, and aminoglycosides [9, 10] rendering the most effective drugs infective, thereby limiting treatment choices for infections.

Although the prevalence of MDR and its development mechanisms in developed countries have been adequately investigated, the evidence on the prevalence of MDR against Enterobacteriaceae in developing countries particularly in sub-Saharan nations is scanty [11]. Given that Ethiopia is a sub-Saharan country, the level of MDR and the prevalence of ESBL-producing Enterobacteriaceae are poorly known. Lack of reliable standardized methods and resources particularly for the detection of ESBL-producing bacteria have been identified as some of the possible explanations.

As the result of considerable variation in the level of MDR and ESBL-producing Enterobacteriaceae in different countries, local epidemiological data along with local resistance patterns are critical for clinicians for the effective management of infections caused by this group of bacteria. To this end, determination of the level of MDR and the prevalence of ESBL production in Enterobacteriaceae in Ethiopia appeared to be one of the top research priorities. Against this background, the aim of the present study was to determine the prevalence of MDR and ESBL production in Enterobacteriaceae by using the VITEK 2 compact system (bioMérieux, France). The VITEK 2 compact system is a fully automated and completely standardized microbiological system that performs both bacterial identification and drug susceptibility testing simultaneously. Apart from precise identification and susceptibility testing, the VITEK ESBL test panel is used for the rapid detection of ESBL production based on simultaneous assessment of the inhibitory effects of cefepime, cefotaxime, and ceftazidime, alone and in the presence of clavulanic acid. The performance of the VITEK ESBL test panel in the detection of ESBL production in Enterobacteriaceae is found out to be concordant with that of the molecular method [12].

## 2. Materials and Methods

### 2.1. Study Site and Period

This study was conducted in the Department of Microbiology and Molecular Biology at Arsho Advanced Medical Laboratory over a period of two years from October 2016 to January 2018. Arsho is the oldest and the largest medical laboratory where patients are referred to culture and drug sensitivity testing. On average, about 50 outpatients per day are referred to the department. Patients having different clinically confirmed disease manifestations agreed to take part in the study, and those with no history of antibacterial drug therapy for not less than two weeks before their attendance were included in the study. History of drug treatment was obtained from physicians' laboratory request form.

### 2.2. Identification and Determination of Antimicrobial Susceptibility

Different clinical samples were collected from patients for culture and susceptibility testing according to standard protocols related to each sample. One clinical sample per patient was collected. Clinical samples were cultured onto primary isolation culture media following standard conventional procedures and incubated at the appropriate temperature, aeration, and period. Pure isolates of bacterial pathogens were preliminarily characterized by colony characteristics and Gram stain. Identification and antimicrobial susceptibility testing of each isolate were performed with the VITEK 2 compact system using the AST-GN72 cards, in accordance with the manufacturer's instructions. Quality control bacteria (*E*. *coli* ATCC 35218 and *K. pneumoniae* ATCC 700603) and pure cultures of bacterial isolates were suspended in 3 ml of sterile saline (aqueous 0.45 to 0.50% NaCl, pH 4.5 to 7.0) in a 12 × 75 mm clear plastic (polystyrene) test tube to achieve a turbidity equivalent to that of a McFarland 0.50 standard (range, 0.50 to 0.63), as measured by the DensiChek (bioMérieux) turbidity meter. These suspensions were used for the inoculation of GN72 identification cards while AST cards were inoculated after bacterial suspensions were further diluted following the instruction of the manufacturer. The definition of Magiorakos et al. [13] was used for categorizing bacterial isolates as multidrug resistance (MDR), nonsusceptibility to at least one agent in three or more antimicrobial categories (XDR), nonsusceptibility to at least one agent in all but two or fewer antimicrobial categories, extensively drug-resistant and pan-drug resistant (PDR), and nonsusceptibility to all agents in all antimicrobial categories.

### 2.3. ESBL Detection

The ESBL phenotype was determined with the VITEK ESBL test panel with six wells containing three third-generation cephalosporins alone (cefepime, cefotaxime, and ceftazidime) and in combination with clavulanic acid (CA) as per the instruction of the manufacturer. Growth in wells having cephalosporin plus CA compared with those containing the cephalosporin alone was assessed by means of an optical scanner. Final results were analyzed using version 4.0 software, an advanced expert system (AES) designed to assess the results produced by the VITEK. The relative reduction in growth in wells having cephalosporin plus CA compared with those containing the cephalosporin alone was considered positive for ESBL production. Strains were noted as ESBL-negative whenever phenotypic interpretations other than ESBLs were suggested by the AES. Quality control strains (*E. coli* ATTC 35218 and *K. pneumoniae* 700603) were included in each run.

#### 2.3.1. Statistical Analysis

All data from the investigation were coded, double-entered, and analyzed using SPSS version 20. Descriptive statistics and logistical regressions were used to estimate crude ratio with 95% confidence interval to the different variables. *P* value <0.05 was considered significant.

#### 2.3.2. Ethics Approval and Consent to Participate

All ethical considerations and obligations were duly addressed. The study was carried out after the approval of the Internal Review Board (IRB) of Arsho Advanced Medical Laboratory private limited company. Data collection was started after obtaining written informed consent from study subjects, and assent form was completed and signed. All the information obtained from the study subjects was coded to maintain confidentiality.

## 3. Results

As depicted in Table 1, of a total of 440 Enterobacteriaceae isolates, 339 (77.1%) were *E. coli* strains, 56 (12.7%) were *K. pneumoniae* strains, and 14 (3.2%) were *P. mirabilis* strains. *Citrobacter freundii and E. cloacae* accounted for 11 (2.5%) each while *S. enterica sub. enterica* accounted for 9 (2.0%).

The antimicrobials tested and percentage antimicrobial resistance profile of Enterobacteriaceae are shown in Table 2. The overall resistance rates of Enterobacteriaceae against cephalosporins ranged from 47.5% for cefepime to 63.2% for cefuroxime. Among the nine cephalosporins tested, cefoxitin was better active with a resistance rate of 22.7%. The overall resistance rates of this group of bacteria against the aminoglycosides, gentamicin and tobramycin, were 31.6% and 32.7%, respectively. Similarly, the overall resistance rate of Enterobacteriaceae was also high against fluoroquinolones, levofloxacin with a resistance rate of 44.5% and ciprofloxacin with a resistance rate of 45.9%. Nitrofurantoin with a resistance rate of 14.3% and piperacillin/tazobactam combination with a resistance rate of 17.3% were better active against this group of bacteria. *E. coli*, the most commonly isolated species, demonstrated less resistance rates against nitrofurantoin (7.7%), piperacillin/tazobactam combination (14.2%), and cefoxitin (15.9%). The resistance rates of the pathogen towards the other antimicrobial agents tested were over 30%. *K. pneumoniae* isolates have high resistance rates against all antimicrobial agents tested. The lowest resistance rate for the bacterium was observed against cefoxitin (i.e., 25%). *C. freundii* and *P. mirabilis* were 100% susceptible to tobramycin while *S*. *enterica sub. enterica* isolates were 100% susceptible to nitrofurantoin and ceftazidime.

The prevalence of MDR isolates of Enterobacteriaceae to 16 antibiotics or antibiotic combinations covering five classes of antimicrobial compounds and ESBL phenotype is shown in Table 3. Out of 440 isolates of Enterobacteriaceae, 185 (42.1%) were MDR of which 151 (34.3%) and 35 (8.95%) were XDR and PDR, respectively. Among 339 strains of *E*. *coli*, 143 (42.2%) were MDR of which 121 (35.7%) and 22 (6.5%) were XDR and PDR, respectively. Of 56 strains of *K. pneumoniae*, 29 (51.8%) were MDR of which 21 (37.5%) and 8 (14.3%) were XDR and PDR, respectively. Out of 11 strains of *C*. *freundii*, 27.3% (3/11) were MDR among which 18.2% and 9% were XDR and PDR, respectively. Similarly, among 11 isolates of *E. cloacae*, 36.4% (4/11) strains were MDR among which 2 (18.2%) were XDR and PDR each.

Of 185 MDR Enterobacteriaceae, 63.9 (118/185) isolates produced ESBLs. 62.2% (89/143) of MDR *E. coli*, 79.3% (23/29) of MDR *K. pneumoniae*, 66.6% (2/3) of MDR *C. freundii*, 75% (3/4) of MDR *E. cloacae*, and 33.3% (1/3) MDR of *P. mirabilis* were producers of ESBL.

## 4. Discussion

In the present study, Enterobacteriaceae were tested against 16 antibacterial agents. Our study demonstrated that the antibiotic resistance pattern of the isolates of Enterobacteriaceae was variable. Nitrofurantoin was the most effective antibiotic with an overall resistance rate of 14.3%. Among drugs of *β*-lactam/*β*-lactamase inhibitor combinations, piperacillin/tazobactam was better active against the isolates with a resistance rate of 17.3%, indicating that ESBLs were sensitive to tazobactam. The isolates were comparatively less susceptible to cephalosporins than other antibiotics. Except cefoxitin with an overall resistance rate of 22.7%, the overall drug resistance rate of the isolates against cephalosporins including the extended beta-lactams was above 45%. High prevalence rate of ESBL-producing isolates noted in this study may be incriminated as one possible explanation for an increase in the level of drug resistance to this category of antibacterial agents. Resistance to *β*-lactams in Enterobacteriaceae is mainly due to the production of *β*-lactamases, which may be encoded either chromosomally or on plasmids [14]. ESBLs are enzymes that have the ability to hydrolyze and cause resistance to various types of newer *β*-lactam antibiotics, including the expanded-spectrum cephalosporins such as cefotaxime, ceftriaxone, and ceftazidime but not to cephamycins such as cefoxitin [3]. Overall drug resistance rates of the isolates against other classes of antibacterial agents such as aminoglycosides (gentamycin and tobramycin) and fluoroquinolones (ciprofloxacin and levofloxacin) in the present study were also high. High antimicrobial resistance rates to these drugs support the observations of Paterson [2] and Pitout et al. [15] who demonstrated that chromosomally or plasmid-encoded ESBLs that are associated with mobile genetic elements carrying genes encode resistance to other antimicrobial agents. High level of drug resistance against fluoroquinolones is of particular interest because it is frequently recommended for the treatment of complicated cystitis in female patients. Fluoroquinolones are also used as a first choice for the treatment of urinary tract infections in men, mainly because of certain advantages of this antibiotic over co-amoxiclav, particularly in terms of its pharmacokinetic properties [16, 17].

The global spread of multidrug-resistant among bacterial pathogens implicated in causing both nosocomial and community-acquired infections is a major threat to public health all over the world [18, 19]. The problem is far important in bacterial species belonging to the Enterobacteriaceae because of their ubiquity in the environment and animal hosts and the relative ease acquisition of plasmids containing genes that encode for ESBLs and other resistance genes that confer resistance to many other classes of antibiotics [3–6]. Multidrug resistance species in Enterobacteriaceae vary from one study to another. In the present study, out of 440 isolates of Enterobacteriaceae, 185 (42.1%) were MDR, among which 151 (34.3%) were XDR and 35 (8.95%) were PDR. The values of MDR and XDR noted in the present study do not substantially deviate from which have formerly been described to be 35% MDR and 12.1% XDR strains, respectively, by Basak et al. [20]. However, a prevalence rate of 96.9% MDR documented by Yadav et al. [21] exceeded the present value by almost three-fold. A similar study conducted by Bajpai et al. [22] revealed 75.8% of the isolates were MDR of which 12% and 2.1% isolates are XDR and PDR, respectively. Extensive exposure of bacteria to antibacterial agents is the main factor promoting the emergence and spread of MDR bacteria. Increased use of over-the-counter antibacterial drugs, incomplete course of therapy, and prolonged therapy for recurrent bacteria diseases are commonly practiced in Ethiopia. These practices could be cited as possible factors for the high prevalence of MDR bacterial species noted in the current study.

Given that Enterobacteriaceae is a large family consisting of many species of Gram-negative bacteria, critical analysis (assessment) of drug resistance and ESBL production for each species within the family appears to be more important than the family in general. To this end, *E. coli* was the most frequent bacterial isolate in the present study, followed by *K. pneumoniae*. This finding was similar to the observation of Sader et al. [23]. Among 185 MDR Enterobacteriaceae isolates, the commonest MDR strains were detected from *Klebsiella pneumonia* (51.8%) followed by *E. coli* (42.2%). Similarly, out of 151 XDR strains isolated, the commonest XDR strains were detected from *K. pneumoniae* (37.5%) followed by *E. coli* (35.7%). The highest PDR strains were also observed from *K. pneumoniae* (14.3%) followed by *E. coli* (6.5%). Our findings were consistent with other previous studies conducted by Aly and Balkhy [24] and Thakur et al. [25]. Higher MDR *E. coli* strains (63%) than the present study were reported by Dehbanipour et al. [26], and 59% of strains were reported by Ali et al. [27]. Similarly, higher MDR strains (80%) of *K. pneumoniae* than the present study were reported by studies conducted in Kenya by Apondi et al. [28] and Wasfi et al. [29] and in Rumania (58%) by Banu et al. [30]. Proteus *mirabilis* that is reported to be the second most common Enterobacteriaceae isolate in European countries by Sader et al. [23], USA by Karlowsky et al. [31], and 3rd in Egypt (77.4%) was the most common isolate in our study. Out of 14 strains of *P. mirabilis*, 3 (21.4%) were MDR of which 2 (14.3%) and 2 (14.3%) were XDR and PDR, respectively. Our finding was consistent with the finding of a similar study conducted in Italy (33.35) by Tumbarello et al. [32]. A study carried out in India by Pal et al. [33], however, demonstrated higher MDR strains of the bacterium in which over 95% of *Proteus* species were found to be MDR and more than 50% were XDR. However, neither the study of Tumbarello et al. [32] nor that of Pal et al. [33] reported PDR strains of *Proteus* species. *Citrobacter* species have become an increasing cause of concern as they are MDR and associated with a higher mortality rate. Misra et al. [34] demonstrated that out of 114 *Citrobacter* species, 63 (52.2%) were found to be MDR. In the present study, out of 11 *C*. *freundii* isolates, 3 (27.34%) were MDR of which 2 (18.2%) were XDR and 9% were PDR. Nontyphoidal salmonellosis caused by *Salmonella enterica* serotypes is a major cause of fatal bacteraemia and infant mortality in sub-Saharan Africa. In this region of Africa, the infection is more prevalent in infants with malaria, HIV, malnutrition, and severe anaemia causing a mortality rate of around 25% [35–37]. Of particular concern is the isolation of multidrug-resistant strain of *S*. *enterica* and development resistance to extended-spectrum *β*-lactam cephalosporins and fluoroquinolone. In the present study, among 9 strains isolated, 33.3% of the strains were MDR and more than 33% of the strains were resistant to ceftriaxone, the drug of choice for treatment of pediatric salmonellosis, and to ciprofloxacin, which is preferable for treatment of adults [38]. Among 11 isolates of *E cloacae*, 36.4% were MDR of which 18.2% of each was XDR and PDR. Our finding was in agreement with the report of Leski et al [10] who reported that out of 15 isolates of the species, about 93.3% were MDR.

MDR strains of ESBL-producing Enterobacteriaceae are of particular concern. This is because of their widespread acquisition of other resistant elements. The phenotypic data generated in the current study, using the Advanced Expert System (AES) in conjunction with the VITEK 2 automated system, indicated a considerably significant prevalence of ESBL producers in the study area, where out of a total of 185 MDR isolates of Enterobacteriaceae, 118 (63.9%) were found to be ESBL producers. Among MDR isolates of each species, 79.3% of *K. pneumoniae*, 75% of *E cloacae*, 66.7% of *C*. *freundii*, 62.2% of *E. coli*, and 33.3% of *P. mirabilis* were ESBL producers. Many countries share a high prevalence of extended-spectrum *β*-lactamase-producing Gram-negative bacteria [39–41]. In summary, given that the majority of therapy for bacterial infection in Ethiopia is empiric and that bacterial pathogens are demonstrating increasing antimicrobial resistance, proper antibiotic policy and measures to restrict the indiscriminative use of cephalosporins, the commonly prescribed drugs in Ethiopia should be taken to minimize the emergence of these multiple *β*-lactamase-producing Enterobacteriaceae.

## 5. Conclusions

The present study demonstrated high prevalence rates of multidrug-resistant and extended-spectrum beta-lactamase-producing Enterobacteriaceae. In order to combat these problems, infection control strategy and proper antibiotic policies should be formulated.

## Figures and Tables

**Table 1:** Number of Enterobacteriaceae isolated from various clinical specimens *n* (%).

Species	Clinical samples							
	Sputum	Urine	Wound	CSF	Ear	Nasal	Blood	Total
*E. coli*	—	269	17	25	8	—	20	339 (77.1)
*K. pneumoniae*	12	25	6	5	—	5	3	56 (12.7)
*C. freundii*	—	5	4	—	2	—	—	11 (2.5)
*P. mirabilis*	—	3	4	—	7	—	—	14 (3.2)
*E. cloacae*		6	1	1	2		1	11 (2.5)
*S. enterica sub. enterica*	—	8	1	—	—	—		9 (2.0)

								
Total	12	316	33	31	19	5	24	440 (100)

**Table 2:** Percentage antimicrobial resistance profile of Enterobacteriaceae against 16 antibacterial agents.

Species	Antimicrobial agents															
	AMC	TZP	CZ	CF	CXMA	CXM	FOX	CPD	CRO	CAZ	PEP	TM	GM	LEV	CIP	FT
*E. coli* (339)	33.9	14.2	50.4	57.8	59.9	60.5	15.9	48.4	47.5	47.2	47.8	34.5	28.9	51.0	51.3	7.7
*K. pneumoniae* (56)	39.3	30.4	71.4	67.9	67.9	66.1	25	66.1	62.5	64.3	64.3	32.1	42.9	26.8	35.7	32.1
*Citrobacter freundii* (11)	54.5	9.1	72.7	72.7	54.5	90.9	72.7	45.5	9.1	9.1	9.1	0	27.3	9.1	18.2	18.2
*P. mirabilis* (14)	7.1	57.1	71.4	78.6	71.4	78.6	72.7	71.4	71.4	71.4	71.4	0	21.4	7.1	7.1	100
*S. enterica sub. enterica* (9)	33.3	0	88.9	88.9	88.9	77.8	77.8	22.2	33.3	0	22.2	77.8	88.9	44.4	33.3	0
*Enterobacter cloacae* (11)	81.8	18.1	81.9	81.9	90.9	72.7	81.9	81.9	27.3	18.1	18.1	18.1	27.3	18.1	18.1	27.3
All isolates (440)	35.5	17.3	55.9	61.4	62.5	63.2	22.7	51.6	48.4	47.5	48.4	32.7	31.6	44.5	45.9	14.3

AMC = amoxicillin/clavulanic acid; CIP = ciprofloxacin; CZ = cefazolin; CXM = cefuroxime; CXMA = cefuroxime axetil; FOX = cefoxitin; CF = cefalotin; CPD = cefpodoxime; CAZ = ceftazidime; CRO = ceftriaxone; PEP = cefepime; GM = gentamicin; LEV = levofloxacin; FT = nitrofurantoin; TZP = piperacillin/tazobactam; and TM = tobramycin.

**Table 3:** Prevalence of multidrug resistance of Enterobacteriaceae against 5 antimicrobial categories and ESBL production.

Species	*R*0	*R*1	*R*2	*R*3	*R*4	*R*5	*n* (%) MDR	*n* (%) (XDR	*n* (%) PDR	*n* (%) ESBL (species)
*E. coli* (339)	93	58	45	49	72	22	143 (42.2)	121 (35.7)	22 (6.5)	89 (62.2)
*K. pneumoniae* (56)	10	10	7	12	9	8	29 (51.8)	21 (37.5)	8 (14.3)	23 (79.3)
*Citrobacter freundii* (11)	—	3	5	1	1	1	3 (27.3)	2 (18.2)	1 (9.0)	2 (66.7)
*P*. *mirabilis* (14)	—	5	6	1	—	2	3 (21.4)	2 (14.3)	2 (14.3)	1 (33.3)
*S*. *enterica sub. enterica* (9)	1	3	2	2	1	—	3 (33.3)	3 (33.3)	—	—
*E. cloacae* (11)	—	1	6	1	1	2	4 (36.4)	2 (18.2)	2 (18.2	3 (75.0)
All isolates (440)	104	80	71	66	84	35	185 (42.1)	151 (34.3)	35 (8.95)	118 (63.9)

*R*0 = no antibiotic resistance; *R*1 = resistant to one antimicrobial category; *R*2 = resistant to two antimicrobial categories; *R*3 = resistant to three antimicrobial categories; *R*4 = resistant to four antimicrobial categories; *R*5 = resistant to five antimicrobial categories.

## Data Availability

The data used to support the findings of this study are included within the article.

## Additional Points

Demographic data and patient history were not documented for this study as the recording of these parameters in the requisition form filled up by physicians was not consistent. Due to resource limitations, molecular methods for the detection of antibiotic resistance mechanisms and types of ESBL genes were not identified.

## References

[1] Nordmann P., Naas T., Poirel L. (2011). Global spread of carbapenemase-producing enterobacteriaceae. *Emerging Infectious Diseases*.

[2] Paterson D. L. (2006). Resistance in gram-negative bacteria: enterobacteriaceae. *The American Journal of Medicine*.

[3] Bradford P. A. (2001). Extended-spectrum beta-lactamases in the 21^st^ century: characterization, epidemiology, and detection of this important resistance threat. *Clinical Microbiology Reviews*.

[4] Ben-Ami R., Rodriguez-Bano J., Arslan H. (2009). A multinational survey of risk factors for infection with extended-spectrum beta-lactamase-producing enterobacteriaceae in non-hospitalized patients. *Clinical Microbiology Reviews*.

[5] Perez F., Van Duin D. (2013). Carbapenem-resistant enterobacteriaceae: a menace to our most vulnerable patients. *Cleveland Clinic Journal of Medicine*.

[6] Brolund A. (2014). Overview of ESBL-producing enterobacteriaceae from a nordic perspective. *Infection Ecology & Epidemiology*.

[7] Moquet O., Bouchiat C., Kinana A. (2011). Class d OXA-48 carbapenemase in multidrug-resistant enterobacteria, Senegal. *Emerging Infectious Diseases*.

[8] Mushi M. F., Mshana S. E., Imirzalioglu C., Bwanga F. (2014). Carbapenemase genes among multidrug resistant gram negative clinical isolates from a tertiary hospital in Mwanza, Tanzania. *BioMed Research International*.

[9] Tada T., Miyoshi-Akiyama T., Dahal R. K. (2013). Dissemination of multidrug-resistant *Klebsiella pneumoniae* clinical isolates with various combinations of carbapenemases (NDM-1 and OXA-72) and 16S rRNA methylases (ArmA, RmtC and RmtF) in Nepal. *International Journal of Antimicrobial Agents*.

[10] Leski T., Vora G. J., Taitt C. R. (2012). Multidrug resistance determinants from NDM-1-producing *Klebsiella pneumoniae* in the USA. *International Journal of Antimicrobial Agents*.

[11] Tansarli G. S., Athanasiou S., Falagas M. E. (2013). Evaluation of antimicrobial susceptibility of enterobacteriaceae causing urinary tract infections in Africa. *Antimicrobial Agents and Chemotherapy*.

[12] Spanu T., Sanguinetti M., Tumbarello M. (2006). Evaluation of the new VITEK 2 extended-spectrum beta-lactamase (ESBL) test for rapid detection of ESBL production in enterobacteriaceae isolates. *Journal of Clinical Microbiology*.

[13] Magiorakos A. P., Srinivasan A., Carey R. B., Carmeli Y., Falagas M. E., Giske C. G. (2011). Multidrug-resistant, extensively drug-resistant and pan-drug resistant bacteria: an international expert proposal for interim standard definitions for acquired resistance. *Clinical Microbiology and Infection*.

[14] Kattel H. P., Acharya J., Mishra S. K., Rijal B. P., Pokhrel B. M. (2008). Bacteriology of urinary tract infection among patients attending Tribhuvan university teaching hospital, Kathmandu, Nepal. *Journal of Nepal Association for Medical Laboratory Sciences*.

[15] Pitout J. D. D., Nordmann P., Laupland K. B., Poirel L. (2005). Emergence of enterobacteriaceae producing extended-spectrum *β*-lactamases (ESBLs) in the community. *Journal of Antimicrobial Chemotherapy*.

[16] Wagenlehner F. M., Weidner W., Naber K. G. (2007). Therapy for prostatitis, with emphasis on bacterial prostatitis. *Expert Opinion on Pharmacotherapy*.

[17] Charalabopoulos K., Karachalios G., Baltogiannis D., Charalabopoulos A., Giannakopoulos X., Sofikitis N. (2003). Penetration of antimicrobial agents into the prostate. *Chemotherapy*.

[18] Anonymous (2013). The antibiotic alarm. *Nature*.

[19] Centers for Disease Control and Prevention (2013). *Antibiotic Resistance Threats in the United States*.

[20] Basak S., Singh P., Rajurkar M. (2016). Multidrug resistant and extensively drug resistant bacteria: a study. *Journal of Pathogens*.

[21] Yadav K. K., Adhikari N., Khadka R., Pant A. D., Shah B. (2015). Multidrug resistant enterobacteriaceae and extended spectrum *β*-lactamase producing *Escherichia coli*: a cross-sectional study in national kidney center, Nepal. *Antimicrobial Resistance and Infection Control*.

[22] Bajpai T., Bhatambare G. S., Pandey M., Meena Varma M. (2014). Prevalence of multi, extensively and pan drug resistant uropathogens among the women patients visiting a tertiary care hospital in central India. *International Journal of Health System and Disaster Management*.

[23] Sader H. S., Biedenbach D. J., Jones R. N. (2003). Global patterns of susceptibility for 21 commonly utilized antimicrobial agents tested against 48,440 enterobacteriaceae in the sentry antimicrobial surveillance program (1997–2001). *Diagnostic Microbiology and Infectious Disease*.

[24] Aly M., Balkhy H. H. (2012). The prevalence of antimicrobial resistance in clinical isolates from gulf corporation council countries. *Antimicrobial Resistance and Infection Control*.

[25] Thakur S., Pokhrel N., Sharma N. (2013). Prevalence of multidrug resistant enterobacteriaceae and extended spectrum *β* lactamase producing *Escherichia coli* in urinary tract infection. *Research Journal of Pharmaceutical, Biological and Chemical Sciences*.

[26] Dehbanipour R., Rastaghi S., Sedighi M., Maleki N., Faghri J. (2016). High prevalence of multidrug-resistance uropathogenic *Escherichia coli* strains, Isfahan, Iran. *Journal of Natural Science, Biology and Medicine*.

[27] Ali I., Rafaque Z., Ahmed S., Malik S., Dasti J. I. (2016). Prevalence of multi-drug resistant uropathogenic *Escherichia coli* in potohar region of Pakistan. *Asian Pacific Journal of Tropical Biomedicine*.

[28] Apondi O. E., Oduor O. C., Gye B. K., Kipkoech M. K. (2016). High prevalence of multi-drug resistant *Klebsiella pneumoniae* in a tertiary teaching hospital in western Kenya. *African Journal of Infectious Diseases*.

[29] Wasfi R., Elkhatib W. F., Ashour H. M. (2016). Molecular typing and virulence analysis of multidrug resistant *Klebsiella pneumoniae* clinical isolates recovered from Egyptian hospitals. *Scientific Reports*.

[30] Banu O., Bleotu C., Burtea M. (2015). Prevalence of multidrug-resistant *Klebsiella pneumoniae* strains isolated from patients with cardiovascular disease. *Journal of Translational Medicine and Research*.

[31] Karlowsky J. A., Jones M. E., Thornsberry C., Friedland I. R., Sahm D. F. (2003). Trends in antimicrobial susceptibilities among enterobacteriaceae isolated from hospitalized patients in the united states from 1998 to 2001. *Antimicrobial Agents and Chemotherapy*.

[32] Tumbarello M., Trecarichi E. M., Fiori B. (2012). Multidrug-resistant *Proteus mirabilis* bloodstream infections: risk factors and outcomes. *Antimicrobial Agents and Chemotherapy*.

[33] Pal N., Sharma N., Sharma R., Hooja J. (2014). Prevalence of multidrug (MDR) and extensively drug resistant (XDR) *Proteus* species in a tertiary care hospital. *International Journal of Current Microbiology and Applied Sciences*.

[34] Misra R., Gandham N., Sardar M. (2012). High prevalence of multidrug-resistant citrobacter species from tertiary care hospital, Pimpri, Pune, India. *Journal of Pharmaceutical and Biomedical Sciences*.

[35] Gordon M. A. (2011). Invasive nontyphoidal salmonella disease. *Current Opinion in Infectious Diseases*.

[36] Bager F., Petersen J. (1991). Sensitivity and specificity of different methods for the isolation of *Salmonella* from pigs. *Acta Veterinaria Scandinavica*.

[37] Morpeth S. C., Ramadhani H. O., Crump J. A. (2009). Invasive non—typhi salmonella disease in Africa. *Clinical Infectious Diseases*.

[38] Guerrant R. L., Van Gilder T., Steiner T. S. (2001). Practice guidelines for the management of infectious diarrhea. *Clinical Infectious Diseases*.

[39] Dugal S., Purohit H. (2013). Antimicrobial susceptibility profile and detection of extended spectrum beta-lactamase production by gram negative uropathogens. *International Journal of Pharmacy and Pharmaceutical Sciences*.

[40] Saied T., Elkholy A., Hafez S. F. (2011). Antimicrobial resistance in pathogens causing nosocomial bloodstream infections in university hospitals in Egypt. *American Journal of Infection Control*.

[41] Mansouri S., Abbasi S. (2010). Prevalence of multiple drug resistant clinical isolates of extended-spectrum beta- lactamase producing enterobacteriaceae in southeast Iran. *Iranian Journal of Medical Sciences*.

